# Amorphous breast calcifications: is BI-RADS 4a appropriate?

**DOI:** 10.1590/0100-3984.2022.0085-en

**Published:** 2023

**Authors:** Tatiane Mendes Gonçalves de Oliveira, Rafael Melo Seksenian, José Galdino Souza Santana, Bárbara Nogueira Caracas de Souza, Felipe Alves de Jesus, Francesca Maia Faria, Larissa Raquel Mouro Mandarano

**Affiliations:** 1 Hospital das Clínicas da Faculdade de Medicina de Ribeirão Preto da Universidade de São Paulo (HCFMRP-USP), Ribeirão Preto, SP, Brazil

**Keywords:** Breast neoplasms, Calcinosis/diagnostic imaging, Calcinosis/pathology, Biopsy/methods, Neoplasias da mama, Calcinose/diagnóstico por imagem, Calcinose/patologia, Biópsia/métodos

## Abstract

**Objective:**

To evaluate the positive predictive value (PPV) of amorphous calcifications and to analyze the imaging variables that could alter the risk of malignancy associated with this finding.

**Materials and Methods:**

This was a retrospective study of 138 stereotactically guided percutaneous vacuum-assisted biopsies of amorphous calcifications, performed between January 2012 and December 2017. All of the patients included were referred for radiological follow-up for a minimum of one year (if the histopathology showed a benign lesion) or for surgical treatment (if the histopathology showed malignancy or a lesion of uncertain malignant potential).

**Results:**

We found that the PPV of amorphous calcifications was 9.42%. However, most of the malignant amorphous calcifications were in cases of invasive carcinoma or high-grade ductal carcinoma in situ, indicating clinically relevant disease. The relative risk of malignancy associated with amorphous calcifications was 6.15 times higher in patients with a family or personal history of breast or ovarian cancer. Neither being postmenopausal nor having dense breasts was found to be predictive of malignancy in patients with amorphous calcifications.

**Conclusion:**

Amorphous calcifications in the breast had a PPV for malignancy of 9.42%, indicating the possibility of placing the finding in subcategory 4a, which requires histopathological analysis. Our finding that the risk of malignancy associated with this subtype of calcifications is up to 6.15 times higher in patients with a family or personal history of breast cancer warrants greater concern regarding the clinical, radiologic, and histopathologic correlations after biopsy.

## Abstract (pt)

**Objetivo:**

Avaliar o valor preditivo positivo (VPP) das calcificações amorfas e possíveis variáveis clínicas e de imagem que possam influenciar no risco de malignidade deste achado de imagem.

**Materiais e Métodos:**

Foram revisados, retrospectivamente, 138 resultados de biópsias percutâneas estereotáxicas a vácuo de calcificações amorfas, entre janeiro de 2012 e dezembro de 2017. Todas as pacientes incluídas apresentavam seguimento radiológico mínimo de um ano (histopatológico benigno) ou tratamento cirúrgico (histopatológico maligno).

**Resultados:**

O VPP das calcificações amorfas foi de 9,42%. As lesões malignas corresponderam predominantemente a carcinomas invasivos, indicando doença clinicamente relevante. O risco relativo de malignidade das calcificações amorfas foi 6,15 vezes maior em pacientes com história familiar ou pessoal de neoplasia de mama ou ovário. *Status* pós-menopausa e mamas densas não foram preditores de malignidade nessas pacientes.

**Conclusão:**

As calcificações amorfas na mama apresentaram VPP de malignidade de 9,42%, sugerindo possibilidade de classificação do achado na subcategoria 4a, com necessidade de investigação histopatológica. Em pacientes com história familiar ou pessoal de câncer de mama, o risco de malignidade deste subtipo de calcificações pode ser até 6,15 vezes maior, justificando maior preocupação na correlação clínica, radiológica e histopatológica após biópsia.

## INTRODUCTION

Calcifications account for 55% of all nonpalpable lesions identified on mammograms. In most cases, calcifications represent benign fibrocystic and proliferative changes in the breast, although they can also be related to lesions of uncertain malignant potential, ductal carcinoma *in situ* (DCIS), or invasive carcinoma^(1,2)^.

The calcifications seen on mammography are evaluated in accordance with the American College of Radiology Breast Imaging Reporting and Data System (BI-RADS), 5th edition^(3)^. Depending on their morphology and distribution, such calcifications are assigned a BI-RADS category (0, 2, 3, 4, 5, or 6), higher numbers indicating a greater risk of malignancy. The morphological descriptor “amorphous” is applied to indistinct, low-density calcifications with a positive predictive value (PPV) of 20% for malignancy, which are therefore defined as BI-RADS category 4b (PPV > 10 and ≤ 50%) and for which histopathological evaluation is recommended^(3)^.

Although the rate of detection of amorphous calcifications has increased with the use of digital mammography and specific medical monitors, the management of this subgroup of calcifications can be challenging. It is not uncommon for amorphous calcifications to be identified only on magnified images, in only one view, or even not defined in the stereotactic window on stereotaxic devices or tables, making it impossible to perform percutaneous biopsy or the preoperative marking process^(4,5,6,7,8,9)^.

Recent studies have shown that the risk of malignancy for amorphous calcifications is low, with reported PPVs below 10%^(10,11)^. Better risk stratification for this mammographic finding could reduce the number of unnecessary biopsies.

The objective of this study was to evaluate the PPV of amorphous calcifications sampled in stereotactically guided percutaneous vacuum-assisted biopsies (VABs), as well as to correlate the variables menopausal status, breast density, and risk factors for breast cancer with the increased risk of malignancy associated with amorphous calcifications.

## MATERIALS AND METHODS

This was a single-center retrospective study, approved by the local research ethics committee. Due to the retrospective nature of the study, the requirement for informed consent was waived. We reviewed the electronic medical records of 431 consecutive patients with a mammographic diagnosis of suspicious calcifications who underwent percutaneous VAB between January 2012 and December 2017. Of the 431 patients identified, 159 had amorphous calcifications without other findings such as nodules, architectural distortions, asymmetries, or suspicious calcifications of other morphologies. Patients who were not in clinical and mammographic follow-up for at least 12 months were excluded, as were those who were diagnosed with a malignant lesion or a lesion of uncertain malignant potential and did not undergo surgical excision. Thus, 26 patients were excluded. Therefore, the study sample comprised 138 lesions, composed exclusively of amorphous calcifications, in 133 women.

### Data analysis and statistical analysis

On routine mammograms of the 133 patients, the craniocaudal and mediolateral oblique views, as well as the magnifications in the craniocaudal and mediolateral/ lateral views, were evaluated by two breast radiologists with two and ten years of experience, respectively, on a specific monitor (RadiForce GX530; Eizo Corporation, Hakusan, Japan), using the BI-RADS lexicon. In cases of disagreement regarding the morphological classification of the calcifications, the images were reviewed jointly by the two radiologists in order to reach a consensus.

Percutaneous VABs were performed on a stereotaxic table (model 3-000a-2400; Lorad Medical Systems Inc., Danbury, CT, USA) and with a vacuum-assisted stereotaxic breast biopsy system (Suros ATEC; Hologic, Marlborough, MA, USA), with a 9G needle. In accordance with the protocol of the facility, at least 12 fragments were removed, after which the fragments collected were X-rayed and a marker clip was placed at the biopsy site. All of the biopsies were supervised by one of the breast radiologists involved in the study.

The histopathological analysis was performed by a pathology team, with double reading, and was obtained by reviewing the electronic medical records. Histopathological diagnoses were divided into benign and malignant. Lesions of uncertain malignant potential (atypical ductal hyperplasia, flat epithelial atypia, lobular carcinoma *in situ*) were classified as benign or malignant according to the histopathological result after surgical excision.

Information on menopausal *status* and risk factors for breast cancer were obtained from electronic medical records. The following factors were considered indicative of a high risk for breast cancer: having a first-degree relative (of any age) with a history of breast or ovarian cancer; carrying a genetic mutation associated with predisposition to breast cancer (such as BRCA1 and BRCA2); and having a risk ≥ 20%, as calculated by the Gail and Claus models. Breast density, classified according to the BI-RADS as composition a, b, c, or d, was obtained by reviewing the mammography reports and was stratified as dense breasts (compositions c and d) or no dense breasts (compositions a and b).

Statistical analysis was performed with Microsoft Excel 2016 and the program R, version 3.6.1 (The R Foundation for Statistical Computing, Vienna, Austria). For the evaluation of categorical variables, the chi-square test and Fisher’s exact test were applied, odds ratios being used in order to determine the relative risk.

## RESULTS

A total of 133 patients were included in the study. The mean age was 54.96 years (range, 37–78 years). Because six patients (8.7%) had two clusters of amorphous calcifications, a total of 138 lesions were evaluated. Of those 138 amorphous calcifications, 100 (72.46%) were completely removed by percutaneous VAB.

Of the 138 lesions evaluated, 125 (90.57%) were classified as benign in the histopathological analysis. The most prevalent diagnoses were usual ductal hyperplasia, in 46 (33.33%); fibrocystic alterations, in 24 (17.39%); benign epithelial proliferations, in 18 (13.04%); fibrosis, in 13 (9.42%); and sclerosing adenosis, in 9 (6.25%).

**Figure 1:**
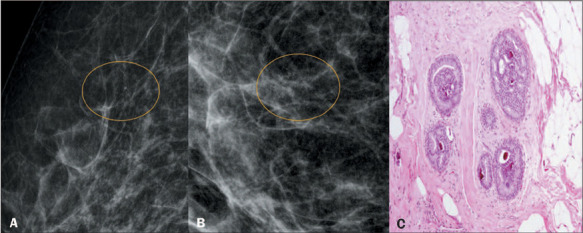
A 65-year-old patient. Magnification mammography in a mediolateral/lateral view (A) and a craniocaudal view (B), showing clusters of amorphous calcifications (circles). C: Histopathology slide showing atypical ductal hyperplasia with a cribriform pattern and intraductal calcifications.

**Figure 2:**
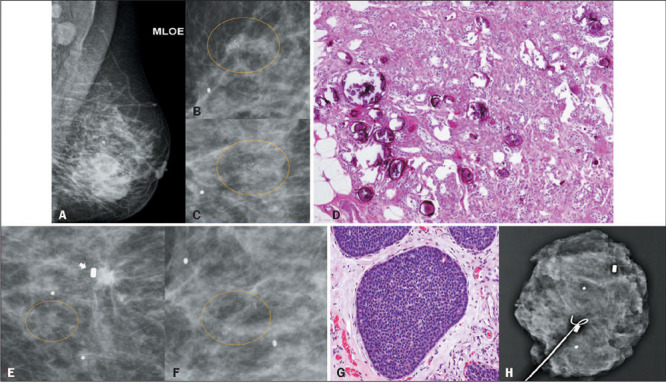
A 58-year-old patient diagnosed with IDC-NOS in the left breast (A). Magnification mammography in a lateral view (B) and a craniocaudal view (C), showing clusters of amorphous calcifications (circles) in the right breast. D: Histopathology slide showing florid adenosis with multiple intraductal and stromal calcifications (hematoxylin and eosin staining; magnification, ×100). Follow-up magnification mammography, performed 23 months later, in a lateral view (E) and a craniocaudal view (F), showing new clusters of amorphous calcifications (circles) near the previous biopsy marker clip (arrow). G: Histopathology slide of a sample obtained in a follow-up percutaneous VAB, showing low-grade DCIS with a solid pattern (hematoxylin and eosin staining; magnification, ×200). H: Radiograph of the surgical specimen showing metallic marker clips from previous biopsies.

Lesions of uncertain malignant potential (Figure 1) were found in eight lesions (5.8%), of which three were atypical ductal hyperplasia, two were flat atypia, and three were lobular carcinoma *in situ* with the classic pattern. After surgical excision, none of those patients were diagnosed with malignancy. One patient in our sample had IDC-NOS in the left breast and a cluster of amorphous calcifications in the right breast with histopathology of florid adenosis and columnar cell changes, without residual calcifications after percutaneous VAB. At 23 months after the biopsy, a new cluster of amorphous calcifications was identified in the right breast, 2.0 cm from the previous biopsy marker clip. A new percutaneous VAB revealed low-grade DCIS (Figure 2). The emergence of this new cluster was considered a new lesion and not an indication of diagnostic underestimation, given the distance from the previous marker clip.

**Figure 3:**
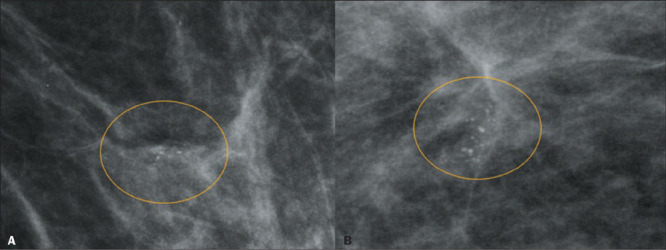
A 67-year-old patient. Magnification mammography in a craniocaudal view (A) and a mediolateral/lateral view (B), showing clusters of amorphous calcifications (circles). Histopathology (not shown) after percutaneous VAB revealed IDC-NOS, Nottingham grade 2, and luminal A molecular classification.

Given that 13 lesions were classified as malignant in the histopathological analysis, the PPV of amorphous calcifications for malignancy was 9.42%. Of the 13 malignant lesions, six (46.15%) were classified as DCIS (one being of high nuclear grade) and seven (53.84%) were classified as invasive ductal carcinoma not otherwise specified (IDC-NOS), predominantly Nottingham histological grades 1 and 2 (Figure 3). Of the seven lesions classified as IDC-NOS, two were luminal A, three were luminal B, one was luminal with no Ki-67 information available, and one showed overexpression of human epidermal growth factor receptor 2.

Eleven patients (8.27%) had risk factors for breast cancer: four had a personal history of breast cancer; six had a family history of breast or ovarian cancer; and one had a risk ≥ 20%, as calculated by the Gail model. For 16 patients (12.03%), the electronic medical records contained no information about risk factors. As shown in Table 1, the relative risk of malignancy for amorphous calcifications in high-risk patients was significant (OR = 6.15; 95% CI: 1.84–30.55).

**Table 1:** Correlations of menopausal status, risk factors for breast cancer, and breast density with the risk of malignancy from amorphous calcifications.

	Biopsy result			
Variable	Benign	Malignant	Total	*P*
Menopausal status				
Postmenopausal	73 (87.95%)	10 (12.05%)	83 (100%)	0.51
In menacme	33 (94.29%)	2 (5.71%)	35 (100%)	
Risk factors for breast cancer				
Yes	7 (63.64%)	4 (36.36%)	11 (100%)	**0.01** *
No	103 (92.79%)	8 (7.21%)	111 (100%)	
Dense breasts				
Yes	60 (88.24%)	8 (11.76%)	68 (100%)	0.52
No	65 (92.86%)	5 (7.14%)	70 (100%)	

* Statistical significance.

Of the 133 women evaluated, 68 (51.12%) were postmenopausal and 35 (26.31%) were in menacme, For the remaining 20 women (15.03%), there was no information regarding menopausal status. The PPV of amorphous calcifications for malignancy was 12.05% among the postmenopausal women and 5.71% among the women in menacme. However, as can be seen in Table 1, the difference between those two groups was not statistically significant (*p* = 0.51).

Of the 138 lesions evaluated, 68 (51.12%) were in women with dense breasts and 70 (52.63%) were in women without dense breasts. As shown in Table 1, there was no statistical correlation between having dense breasts and the risk of malignancy for amorphous calcifications (*p* = 0.52).

## DISCUSSION

According to the BI-RADS, amorphous calcifications have a PPV for malignancy of 20%^(3)^. Other studies^(1,12,13,14)^ have found a PPV between 15% and 29%. However, none of those studies were aimed at specifically evaluating amorphous calcifications and therefore had smaller samples of patients showing that feature. In our study, the PPV of amorphous calcifications was 9.42%. Similar PPV values—7.9%, 7.1%, 10.3%, and 10.5%—were reported, respectively, by Kim et al.^(6)^, Metaxa et al.^(7)^, Oligane et al.^(2)^, and Ferreira et al.^(8)^. This risk stratification brings amorphous calcifications closer to BI-RADS category 4a than category 4b (as currently suggested). Iwase et al.^(11)^ obtained an even lower PPV for amorphous calcifications (2.8%), suggesting the possibility of mammographic follow-up as an alternative to biopsy. However, in their study, histopathological confirmation was obtained in only 29.6% of the patients and calcification clusters that had remained stable for at least 24 months were considered benign. Making that assumption could have resulted in underestimation of the number of malignant lesions with growth that is more insidious, such as DCIS.

Although a malignancy risk of 9.42% for amorphous calcifications suggests that the PPV of this finding for malignancy is lower than is currently accepted, it is still recommended that biopsy and histopathological evaluation be performed. In the present study, the majority (53.84%) of malignant amorphous calcifications were invasive carcinomas, indicating clinically relevant disease. That finding is in contrast with those of other studies in the literature, which have shown a predominance of carcinomas *in situ* among malignant calcified lesions^(2,4,5,8,9,12,13)^. That discrepancy could be explained, in part, by the fact that malignant lesions accounted for only a small proportion (9.42%) of the lesions evaluated in our study. In addition, of the seven lesions diagnosed as invasive carcinoma, six (85.7%) had occurred in patients with dense breasts. Dense breasts reduce mammographic sensitivity, and the superimposition of the dense parenchyma could have prevented the identification of nodules and distortions, making the calcifications the most evident finding.

Of the eight lesions of uncertain malignant potential in our sample, none were diagnosed as malignant after surgical excision. In addition, none of the patients with DCIS were diagnosed with invasive cancer after surgery. Furthermore, among the patients diagnosed with benign lesions, there was no progression of residual calcifications during the clinical follow-up period.

According to Philpotts et al.^(15)^, the rate of underestimation by percutaneous VAB with an 11G needle is 16.3% for all calcification subtypes. We used 9G needles, removing at least 12 fragments, thus obtaining larger samples of breast tissue, as previously demonstrated^(11,13,16)^. In our sample, excision of all calcifications was achieved in 100 (72.46%) of the 138 lesions biopsied, in five (62%) of the eight lesions of uncertain malignant potential, and in 11 (84.6%) of the 13 malignant lesions. As shown in previous studies^(11,13,15,16)^, the larger tissue samples and the high rate of complete excision could explain the low rate of diagnostic underestimation in our study.

We found that the number of amorphous calcifications was comparable between the patients with and without dense breasts (68 and 70 lesions, respectively). Although most (61.53%) of the malignant lesions identified were in women with dense breasts, there was no statistical correlation between the PPV of amorphous calcifications and having dense breasts, as previously reported by Berg et al.^(13)^.

In the present study, the PPV of amorphous calcifications for malignancy was higher among the postmenopausal women than among the women in menacme (12.05% vs. 5.71%), although the difference was not statistically significant. Oligane et al.^(2)^ found that the PPV of amorphous calcifications was 3.1% in women under 50 years of age with no family history of breast cancer, whereas Grimm et al.^(14)^ found it to be 25% in women over 70 years of age. Those findings suggest that age is correlated with the risk of malignancy in patients with amorphous calcifications. The small size of our sample and our decision to evaluate the parameter menopausal status, which indirectly infers age, could partially explain this disagreement between our data and those of previous studies.

We found that the risk of malignancy for amorphous calcifications was 6.15 times greater in the patients at high risk for breast cancer, translating to a PPV of 36.36%. Other authors have reported a correlation between a personal history of breast or ovarian cancer and an increased risk of malignancy in patients with amorphous calcifications^(2,7,13)^.

Limitations of our study include a selection bias due to its retrospective nature and the small sample size, as well as a relatively short (12-month) clinical and imaging follow-up period after percutaneous VAB in 17.29% of the patients. That short follow-up period, albeit in only a small fraction of the sample, could have resulted in underestimation of the diagnosis of slow-growing low-grade DCIS, which can be identified only 24 months after biopsy. In addition, we did not include the statistical evaluation of the distribution of calcifications as a predictor of malignancy already defined by the BI-RADS, because in our sample only 14 cases had a linear/segmental distribution, which would not result in statistical significance. However, we included only amorphous calcifications, excluding any other calcification morphology and associated imaging findings that could interfere with the assessment of the risk of malignancy. Furthermore, there have been few studies correlating the risk of malignancy of amorphous calcifications with variables related to breast cancer risk, such as menopausal status, breast density, and a personal or family history of breast cancer.

## CONCLUSION

Amorphous calcifications in the breast presented a PPV for malignancy of 9.42%, which suggests the possibility of classifying the finding as BI-RADS subcategory 4a, allowing a better radiological-histopathological correlation, as well as facilitating the follow-up of benign lesions and lesions of uncertain malignant potential. In patients with a family or personal history of breast cancer, the risk of malignancy from this subtype of calcifications can be up to 6.15 times greater, justifying greater concern in the radiological-histopathological correlation after biopsy. Being postmenopausal and having dense breasts do not appear to be predictors of malignancy in women with amorphous calcifications.
